# Catastrophic Antiphospholid Syndrome – An Unusual Case Report

**DOI:** 10.5005/jp-journals-10071-23180

**Published:** 2019-06

**Authors:** Sneha Madkaiker

**Affiliations:** Critical Care Medicine, Lilavati Hospital and Research Centre, Mumbai, Maharashtra, India

**Keywords:** Catastrophic antiphospholipid syndrome, Cerebral infarcts, Lupus anticoagulant, Purpura, Renal failure

## Abstract

Antiphospholipid syndrome (APLS) is characterised by venous or arterial thrombosis and/or adverse pregnancy outcome in the presence of persistent laboratory evidence of antiphospholipid antibodies. Catastrophic Antiphospholipid Syndrome (CAPS) is a severe and rare form of antiphospholipid syndrome characterised by multiple site thrombosis involving small, medium and large blood vessels occurring over a short period of time (usually 1 week) causing multiorgan failure.

We present an unusual case of left upper limb acute arterial thrombosis with purpura fulminans like skin lesions precipitated by swine flu (H1N1) infection with adult respiratory distress syndrome subsequently developing acute renal failure, retinal infarcts, multiple acute cerebral infarcts, cardiac valvular vegetations and hemolytic anemia with recurrent bleeding episodes. A positive lupus anticoagulant confirmed the diagnosis of CAPS. In spite of early initiation of triple therapy (anticoagulation, high dose steroids, plasmapheresis) our patient did not survive. This rare case of probable CAPS is presented with an aim to study the clinical manifestations, laboratory findings, efficacy of therapy and prognosis in the medical ICU.

**How to cite this article:** Madkaiker S. Catastrophic Antiphospholid Syndrome – An Unusual Case Report. Indian J Crit Care Med 2019;23(6):276–280.

## INTRODUCTION

Antiphospholipid syndrome (APLS) also called Hughes syndrome is a heterogenous autoimmune disorder of hypercoagulation, manifested mostly as arterial and venous thrombosis, recurrent fetal loss, thrombocytopenia and by the presence of aPL antibodies such as anticardiolipin antibodies and lupus anticoagulant.^1,2^ Catastrophic antiphospholipid syndrome (CAPS) also known as Asherson's syndrome is a rare and fatal form of APLS resulting in multiorgan falure. Although CAPS develops in 1% of the patients with APLS, it is associated with a mortality as high as 50%.^3–5^ APLS occurs either as a primary or a secondary condition in the setting of an underlying autoimmune disease usually systemic lupus erythromatosis (SLE).^1,4,6^ Antiphospholipid antibodies such as lupus anticoagulant and antibodies against cardiolipin and β2 glycoprotein are serological hallmarks of CAPS.^4,6,7^

Since it is associated with high morbidity and mortality^5^, early diagnosis and an aggressive therapy seems to be the best treatment option.^6,7^

Here we present a case of probable CAPS presenting as an acute arterial thrombosis with skin involvement and rapidly progressing to acute renal failure, acute ischemic cerebral infarcts, retinal infarcts, cardiac valvular vegetations and hemorrhagic manifestations precipitated by a viral infection.

## Case

A 45-year-old female, hyperthyroid since 10 years, presented to us with fever and chills since 7 days, cough and throat pain since 4 days and progressive breathlessness since 2 days. She was treated at a local hospital as viral interstitial pneumonitis with acute respiratory distress syndrome (ARDS) and shifted to our hospital in view of worsening hypoxia. On examination, she was afebrile, warm, pulse 110 beats per minute, blood pressure (BP) 140/90 mm Hg, SpO_2_ 75% on 15L O_2_, with visible respiratory distress. She was conscious, alert and neurologically intact. Air entry was equal on both sides, with bilateral diffuse crepts while other systemic examination was unremarkable.

**Fig. 1:**
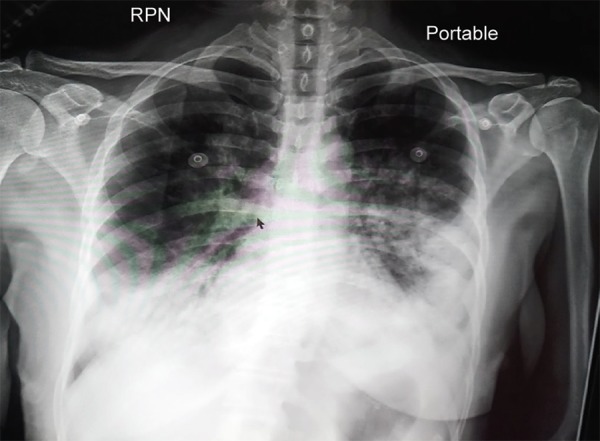
Xray chest on presentation 1 showing bilateral infiltrates

ECG was normal, X-ray chest showed bilateral infiltrates (Fig. 1), Arterial blood gas with pH7.42/ pCO_2_30.9/ pO_2_61.7/ HCO_3_20/ Sat 88.9% on 60% FiO_2_ noninvasive ventilation. She was initiated on intravenous meropenem, tab clarithromycin, tab oseltamivir and supportive treatment. In view of persistent hypoxia patient was intubated and ventilated during which she required a low dose of noradrenaline. Left radial artery was cannulated for BP monitoring. Initial lab test showed a hemoglobin 8 g/dL, total counts 5560/cu mm, platelet 1.73 lakh/cu mm with normal coagulation profile, normal renal and liver functions and procalcitonin of 0.5U. Transthoracic 2Decho was normal with an ejection fraction of 50%, no regional wall abnormalities and normal valves.

**Fig. 2:**
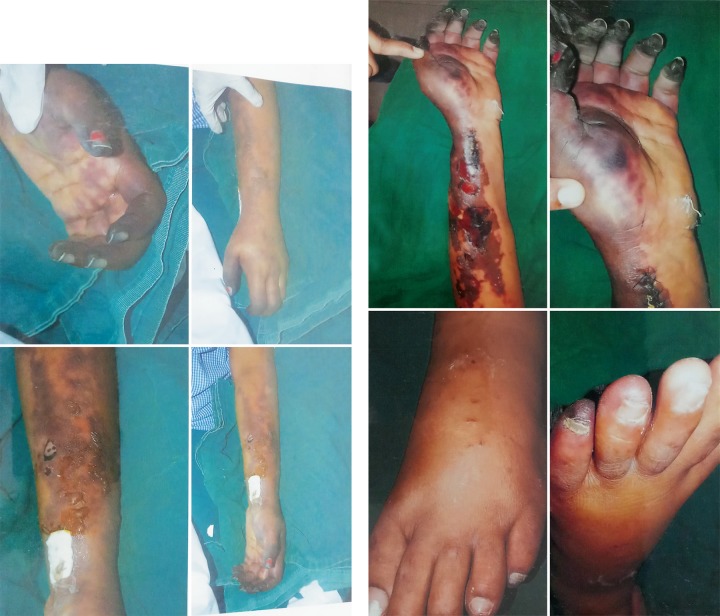
Digital ischemia with forearm and hand skin changes

On day two evening, there was no back flow in the arterial line and hence repositioned to left dorsalis pedis artery. Next morning, the patient developed deep cyanotic changes in all the finger tips of left upper limb with patchy ecchymotic skin lesions involving the whole forearm with no radial pulsations (Fig. 2). Urgent left upper limb arterial doppler showed an echogenic thrombus in left radial artery from mid forearm to wrist. Urgent left radial embolectomy was performed. Post procedure radial pulsations were well felt. But on the next day, radial pulsations were lost again and her left fingers showed features of severe gangrene with blistering and extensive ecchymotic changes involving the whole forearm and hand. Due to irreversible vascular thrombosis, it was decided to go conservative and hence injection enoxaparin 40 mg subcutaneous twice a day was continued.

Over the next few days, although the PaO_2_/FiO_2_ ratio improved, PEEP was reduced and noradrenaline tapered off, patient became febrile and drowsy. Dose of meropenem increased to 2 gm 8 hourly and linezolid added pending cultures. Her throat swab was positive for swine flu (HINI) virus, thyroid stimulating hormone (TSH) ≤ 0.002 U/dL. Neomercazole was reintroduced. Subsequently patient developed hypertension which required nitroglycerine infusion. Serum creatinine increased to 2.65 mg/dL. Urine routine showed significant proteinuria and hematuria. Urine culture grew *Enterococcus feacium* which was covered with appropriate antibiotics. Blood cultures were negative. Fundoscopy revealed retinal infarcts raising suspicion of vasculitis. Patient developed pervaginal bleeding although platelet count and coagulation was normal. Ultrasound pelvis revealed multiple uterine fibroids.

**Fig. 3:**
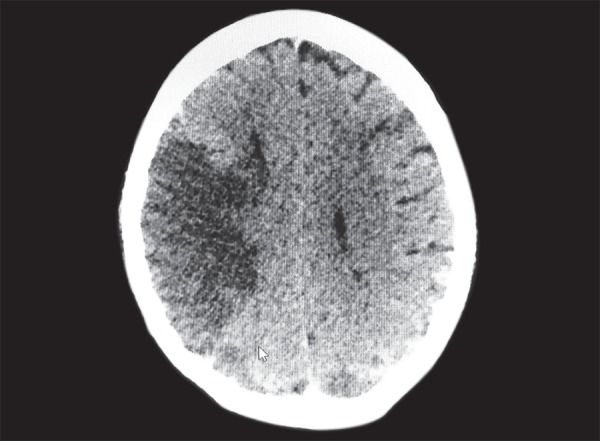
CT brain showing large MCA infarct with multiple small infarcts at other sites

**Fig. 4:**
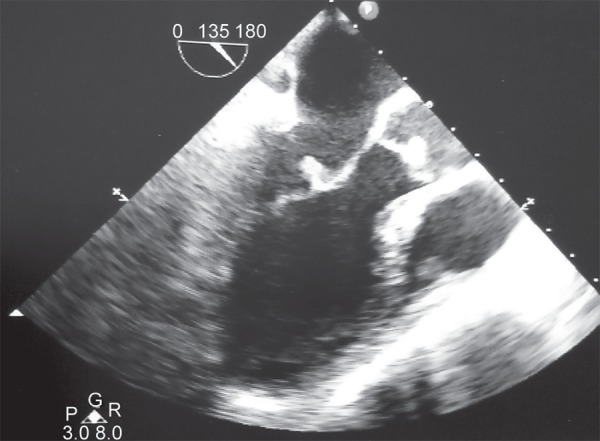
2D echo short axis view showing ball like vegetations on mitral and aortic valve

**Fig. 5:**
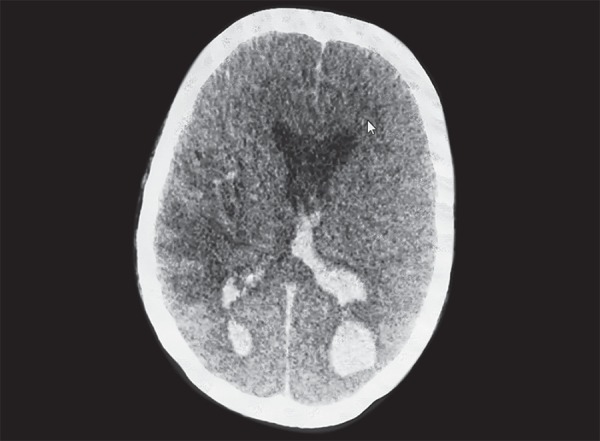
CT brain showing intracerebral bleed with ventricular extention

She continued to have fever and obtunded sensorium with stable hemodynamics and a better gas exchange but with severe dry gangrenous changes of the left upper limb. We concluded that the source could be the gangreneous upper limb with secondary infection so antibiotics were escalated and planned for amputation once patient was stable. Her serum creatinine increased to 4.6 mg/dL although the urine output was maintained. CT brain done as a part of the work up revealed a large acute right MCA infarct with minimal midline shift and few small infarcts in the left parietal, left temporal, B/L occipital lobes, and right cerebellar hemisphere with no evidence of hemorrhage (Fig. 3). Dual antiplatelets were started. Antinuclear antibodies, anti double stranded DNA antibodies, Antineutrophil cytoplasmic antibodies were negative and C3, C4 was normal. A repeat 2 Decho revealed nodular echogenic shadows on anterior mitral leaflet and on right coronary cusps with preserved left ventricular function which was later confirmed on transesophageal echocardiography (Fig. 4). So an acute left radial artery thrombosis with gangrene of left upper limb, probably embolic or thrombotic phenomenon with vegetations on mitral and aortic valves probably mirantic, multiple non watershed cerebral infarcts, in H1N1 positive patient suggested a vasculitic process with gangrene triggered by H1N1 viral infection.

Patient continued to have a small persistant pervaginal bleed but this time associated with progressively worsensing thrombocytopenia. On workup her peripheral smear showed a few schistocytes with a direct coomb test positive. Her serum ADAMTS-13 was sent and a diagnosis of thrombotic thrombocyptopenic purpura (TTP) was contemplated and plasmapheresis was decided. Subsequently her lupus anticoagulant was positive, protein C and protein S were low and factor V Leiden was also positive. Plasmapheresis and hemodialysis were initiated in view of catastrophic antiphospholipid syndrome and worsening acute kidney injury with anuria. Since there was no improvement seen in the neurological status and with worsening metabolic acidosis, recurrence of fever, anuria, intermittent PV bleeding, worsening thrombocytopenia, repeated negative blood, urine and endotracheal cultures and coagulopathy despite five sessions of plasmapheresis, high dose steroids were initiated and only clopidogrel was continued while anticoagulation was already stopped.

After two weeks of ventilation, tracheostomy was planned but she developed a sudden massive drop in hemoglobin to 6.6 g/dL on the same day. On neurological examination she was comatosed with fixed and dialated pupils. Urgent CT brain showed a large intraparenchymal bleed in left occipital region with perilesional edema with extension in both lateral, 3rd and 4th ventricles and bilateral extensive subarachanoid hemorrhage (Fig. 5).

Prognosis was explained to the relatives. In view of irreversible neurological damage they took a decision of withdrawal of care. Patient sustained cardiac arrest after two days.

## DISCUSSION

Antiphospholipid antibody syndrome is a heterogeneous multisystem autoimmune disorder of hypercoagulation^1^. It is regarded as primary or secondary if there are other associated connective tissue disorders like SLE.^1,3,6^ It is predominantly seen in females, with a female to male ratio of 3.5:1.^1,6^ Catastrophic antiphospholipid syndrome, first described by Asherson R. in 1992, is a fatal variant of APLS occurring in 1% of APLS patients.^3–6^

Putative pathogenetic mechanisms for APLS can be arbitraily divided into four interrelated groups which include cellular activation, inhibition of anticoagulation, inhibition of fibrinolysis and complement activation.^7^ The development of thrombi and the high risk of recurrent thrombotic events has been linked to the high titres of lupus anticoagulant and anticardiolipin antibodies^1^.

Our patient was a 45-year-old female, with no underlying SLE or other autoimmune disorders, suggesting possibility of a primary CAPS. But subsequently she was positive for factor V Leiden with deficient protein C and protein S so an underlying prothrombotic state could not be ruled out. As per the CAPS registry created in the year 2000 of 280 patients, CAPS is commonly found in females (72%) with a mean age of 37 ± 14 years with majority suffering from primary APLS (40%).^6,7^ Interestingly CAPS was the first manifestation of APLS in 46% of patients, as in our case.^4^ Concurrent autoimmune disease like SLE is seen in 40%, SLE like syndrome in 5% and other autoimmune pathologies in 9%.^7^ Triggering factors leading to CAPS are infection(22%), surgery(10%), withdrawal of anticoagulation(8%), medication(7%), obstetric complication, neoplasm, and lupus flare.^4,6–9^ In our case H1N1 viral infection was a precipitating factor for the thrombotic microangiopathic state.

The diagnosis of APLS is based on the clinical features (depending on the organ involved by thrombosis) and the laboratory criteria.^10–12^ The major organs involved during catastrophic episodes were renal (71%), lungs (64%), brain (62%), heart (51%), and skin (50%), liver and Gastrointestinal tract as per CAPS registry.^4,6,7^

Our patient developed left upper limb ischemic skin changes in all digits which was due to acute arterial thrombosis and progressed to digital gangrene and purpura fulminans like skin lesions. Cutaneous involvement in CAPS includes livedo reticularis, acrocyanosis, purpura, ecchymosis, splinter hemorrhages and necrosis resulting in ulcerations.^7,13^ Renal involvement is defined by ≥50% increase in creatinine concentration, proteinuria of > 0.5gms/day, severe hypertension (BP >180/100) or combination of these.^8^ Renal infarction is rare.^5,7^ In our case there was a sudden increase in serum creatinine with proteinuria and hypertension. Lung involvement includes ARDS, pulmonary embolism and rarely alveolar hemorrhages.^1,7^ Common CNS presentations being hypertensive encephalopathy, ischemic encephalopathy, stroke, seizure, cortical venous thrombosis and altered mental status.^6,7^ Our patient also developed an acute worsening of sensorium due to a large MCA and other territorial multiple infarcts including cerebellum progressing terminally to intracerebral bleed. Cardiac involvement presents as coronary thrombosis causing unstable angina and myocardial infarction, valvular regurgitant lesions, and valvular sterile vegetations.^1,6,7^ In our patient we also observed fluffy ball like vegetations involving the anterior mitral leaflet and the aortic cusps with no regional wall motion abnormality. Ocular involvement is rare with only 5.8% of the cases reporting retinal infarcts and choroidal thrombosis.^4,7^ A rare case of bilateral central retinal artery occlusion with profound vision loss has been reported.^7^ Retinal infarcts was the first hint for an underlying vasculitic process in our patient. Hematological involvement forms the most important part of the clinical spectrum of CAPS with thrombotic and hemorrhagic manifestations occurring concurrently most of the time and was also seen in our patient.^7,14^ Thrombotic microangiopathy and small vessel occlusive disease^6,7^, a pathological hallmark of CAPS could not be demonstrated on histology in our patient due to overall poor condition.^6,7^

Laboratory findings in CAPS include thrombocytopenia, hemolytic anemia which is often accompanied by small number of schistocytes in about 14% and disseminated intravascular coagulation.^1,3,4,7,14^ These are also the hematological manifestations of CAPS. The autoantibodies to diagnose APLS are anti β_2_ glycoprotein-1, anticardiolipin antibody and lupus anticoagulant.^1,6,7^ The 2006 revised classification criteria for APLS updated the time frame for the presence of elevated titres of aPL antibodies from 6 weeks to 12 weeks.^1,6,11^ In our patient we demonstrated haemolytic anaemia with peripheral schistocytes, thrombhocytopenia and lupus anticoagulant which could not be repeated due to early death of our patient.

### Updated Antiphospholipid Syndrome Classification Criteria^10–12^

#### Clinical Criteria

- Vascular thrombosis:
  ≥1 clinical episodes of arterial, venous, or small vessel thrombosis, in any tissue or organ
- Pregnancy morbidity:
  – ≥1 unexplained deaths of a morphologically normal fetus at or beyond the 10th week of gestation, or
  – ≥1 premature births of a morphologically normal neonate before the 34th week of gestation because of: eclampsia, severe preeclampsia, or recognized features of placental insufficiency, or
  – ≥3 unexplained consecutive spontaneous abortions before the 10th week of gestation, with maternal anatomic or hormonal abnormalities and paternal and maternal chromosomal causes excluded.

#### Laboratory Criteria

- Lupus anticoagulant present in plasma, on ≥2 occasions at least 12 weeks apart
- Anticardiolipin antibody of IgG and/or IgM isotype, in medium or high titer (>40 GPL or MPL, or > the 99th percentile), on ≥2 occasions, at least 12 weeks apart.
- Anti-β2-glycoprotein-I antibody of IgG and/or IgM isotype, in medium or high titer (> the 99th percentile), on ≥2 occasions, at least 12 weeks apart.

Definite APS is present if at least one of the clinical criteria and one of the laboratory criteria are met.

### Preliminary Classification Criteria for Catastrophic Antiphospholipid Syndrome^10–12^

- Evidence of involvement of three or more organs, systems and/or tissues
- Development of manifestations simultaneously or in less than a week
- Confirmation by histopathology of small-vessel occlusion^*^Vasculitis may coexist, but significant thrombosis must be present as well.
- Laboratory confirmation of the presence of antiphospholipid antibodies^†^“Positive aPL” twice 12 weeks apart (of note, the original Sapporo APS classification criteria required two positive aPL tests 6 weeks apart, which has been changed to 12 weeks as part of the updated Sapporo APS classification criteria.

#### Definite Catastrophic Antiphospholipid Syndrome

All four criteria present

#### Probable Catastrophic Antiphospholipid Syndrome

- All four criteria, except only two organs, systems, and/or tissues involved
- All four criteria, except for the absence of laboratory confirmation of antiphospholipid antibodies
- Criteria 1, 2, and 4
- Criteria 1, 3, and 4, with the development of a third event more than 1 week but within 1 month of presentation, despite anticoagulation

Histologically CAPS is characterised by acute thrombotic microangiopathy.^6,7^ Hence CAPS should be differentiated from other thrombotic microangiopathies such as hemolytic uremic syndrome, thrombotic thrombocytopenic purpura (TTP), disseminated intravascular coagulopathy, heparin induced thrombocytopenic thrombosis and severe sepsis.^4,8^ Sepsis and TTP were the differential diagnosis in our patient. However, it should be noted that her ADAMTS 13 activity was negative and her lupus anticoagulant was positive with persistently negative cultures.

CAPS is life threatening systemic disease and is associated with high morbidity and mortality.^1,3,6,7^ Therefore aggressive multidisciplinary collaborative treatment strategy is indicated. A combination of anticoagulation and antiplatelets, high dose steroids, IVIg or plasma exchange, cyclophosphamide and the latest Eculizumab has been found to be effective.^3,6,7,11,12^ Our patient did receive the triple therapy in the form of therapeutic enoxaparin, dual antipletelets, plasmapheresis and high dose methylprednisolone, but the disease progression was so rapid and simultaneous that she ultimately succumbed to massive intracranial hemorrhage within three weeks of admission. But the recent studies have shown a significant decline in the mortality rate in CAPS from a high of 53–33% which is attributed to early diagnosis and the rapid initiation of the aforementioned treatment strategy in combination.^7,15^

## CONCLUSION

CAPS patient suffer from a life threatening acute multiple organ thrombosis, usually accompanied by microthrombosis and hematoloical manifestations with high titre of antiphospholipid antibodies. In our patient the occurrence of so many manifestations simultaneously over a short span confirmed the diagnosis of catastrophic APLA syndrome. Without early diagnosis and treatment, prognosis is poor. It is important to anticipate CAPS and CAPS like disease in young patients with multiorgan involvement and overlapping other thrombotic microangiopathies and known triggering factors in order to halt the mortality and improve prognosis.
